# Loss of RAB39B does not alter MPTP-induced Parkinson’s disease-like phenotypes in mice

**DOI:** 10.3389/fnagi.2023.1087823

**Published:** 2023-01-25

**Authors:** Zijie Wang, Dingting Yang, Yiru Jiang, Yong Wang, Mengxi Niu, Chong Wang, Hong Luo, Huaxi Xu, Jingwen Li, Yun-wu Zhang, Xian Zhang

**Affiliations:** ^1^Fujian Provincial Key Laboratory of Neurodegenerative Disease and Aging Research, School of Medicine, Center for Brain Sciences, The First Affiliated Hospital of Xiamen University, Institute of Neuroscience, Xiamen University, Xiamen, China; ^2^Department of Neurosurgery, Xiang’an Hospital of Xiamen University, Xiamen, China; ^3^Department of Basic Medical Sciences, School of Medicine, Xiamen University, Xiamen, China

**Keywords:** MPTP, RAB39B, Pakinson’s disease, mouse behavior, dopaminergic neurons

## Abstract

Parkinson’s disease (PD) is a common neurodegenerative movement disorder with undetermined etiology. A major pathological hallmark of PD is the progressive degeneration of dopaminergic neurons in the substantia nigra. Loss-of-function mutations in the *RAB39B* gene, which encodes a neuronal-specific small GTPase RAB39B, have been associated with X-linked intellectual disability and pathologically confirmed early-onset PD in multiple families. However, the role of RAB39B in PD pathogenesis remains elusive. In this study, we treated *Rab39b* knock-out (KO) mice with MPTP to explore whether RAB39B deficiency could alter MPTP-induced behavioral impairments and dopaminergic neuron degeneration. Surprisingly, we found that MPTP treatment impaired motor activity and led to loss of tyrosine hydroxylase-positive dopaminergic neurons and gliosis in both WT and *Rab39b* KO mice. However, RAB39B deficiency did not alter MPTP-induced impairments. These results suggest that RAB39B deficiency does not contribute to PD-like phenotypes through compromising dopaminergic neurons in mice; and its role in PD requires further scrutiny.

## 1. Introduction

Parkinson’s disease (PD) is the second most common central neurodegenerative disease after Alzheimer’s disease (von Campenhausen et al., 2005; Tysnes and Storstein, 2017). PD affects about 1–2% of the population over the age of 60 and about 5% of the population over the age of 85 (Van Den Eeden et al., 2003; de Lau and Breteler, 2006; Masato et al., 2019). With an increase in population aging worldwide, PD has been causing serious social and economic burden. The clinical diagnosis of PD is based on the motor symptoms, including bradykinesia, muscular rigidity, rest tremor, and postural and gait impairment (Gibb and Lees, 1988; Gelb et al., 1999). The pathologic hallmarks of PD are progressive loss of dopaminergic neurons in the substantia nigra pars compacta (SNpc) and presence of intracellular inclusions of aggregated α-synuclein called Lewy bodies (LBs). In addition, neuroinflammation plays an important role in the progression of PD. Activated microglia and release of toxins may be involved in the process of neurodegeneration (Hirsch and Hunot, 2000). The environmental and genetic causes have long been considered important risk factors for PD, but the exact etiology of PD remains unknown.

The *RAB39B* gene consists of two exons spanning 3,764 bp of human genomic DNA on the X chromosome and encodes a neuronal-specific protein named RAB39B which is localized in the Golgi compartment (Stankovic et al., 1997; Cheng et al., 2002; Giannandrea et al., 2010). As a small GTPase, RAB39B primarily plays a role in intracellular vesicle trafficking (Chavrier et al., 1990; Cheng et al., 2002). Mutations in *RAB39B* gene are associated with a variety of neurological disorders including X-linked intellectual disability (XLID), autism, epilepsy, macrocephaly and early-onset Parkinson’s disease (EOPD) (Giannandrea et al., 2010; Wilson et al., 2014; Mata et al., 2015; Shi et al., 2016; Woodbury-Smith et al., 2017; Gao et al., 2020).

A loss-of-function mutation in *RAB39B* was originally found in pathologically confirmed EOPD who have extensive dopaminergic neuron loss in substantia nigra and widespread Lewy body pathology (Wilson et al., 2014). Subsequently, additional *RAB39B* mutations associated with EOPD have been reported. Most of these mutations result in complete loss of RAB39B (Lesage et al., 2015; Güldner et al., 2016; Shi et al., 2016; Ciammola et al., 2017; Woodbury-Smith et al., 2017), and some of them lead to subcellular mislocalization of RAB39B (c.574G > A; p.G192R) (Mata et al., 2015) and dysregulated protein homeostasis (c.503C > A; p.T168K) (Wilson et al., 2014; Gao et al., 2020). However, *RAB39B* mutations were not detected in several large-scale cohort screenings of familial PD, indicating that *RAB39B* mutations might be rare in PD (Yuan et al., 2015; Hodges et al., 2016; Löchte et al., 2016; Lin et al., 2017). Some studies have demonstrated that RAB39B is involved in the regulating the homeostasis, oligomerization and aggregation of α-synuclein *in vitro* (Wilson et al., 2014; Gonçalves et al., 2016), but the underlying mechanism is largely unclear.

In our previous study, we found that *Rab39b* KO mice predominately exhibited impairments of learning and memory and synaptic plasticity, whereas their motor behavior impairment was not obvious (Niu et al., 2020). Since PD is considered to be the result of a complex interaction of multiple risk factors, including genetic factors, environmental factors, age, sex, and other factors (Kalia and Lang, 2015), it is possible that the effect of RAB39B deficiency on PD requires the involvement of other factors. In this study, we induced PD-like phenotypes including motor deficits and dopaminergic neuron degeneration in wild type and *Rab39b* KO mice by MPTP, and explored whether RAB39B deficiency could alter MPTP-induced impairments.

## 2. Methods

### 2.1. Animals

C57BL/6 J *Rab39b* knock-out (KO) mice were generated and genotyped as previously described (Niu et al., 2020). C57BL/6 J wild type (WT) mice were provided by Xiamen University Laboratory Animal Center. Mice were kept at 20–25°C with a 12 h light/dark cycle and with free access to food and water. Male *Rab39b* KO or wild-type (WT) littermates at 9–15 weeks of age were subjected to the experiments. All procedures and protocols involving animals were performed in accordance with the guidelines of the National Institutes of Health Guide for the Care and Use of Laboratory Animals, and were approved by the Animal Ethics Committee of Xiamen University (XMULAC20170207).

### 2.2. MPTP administration

**Figure 1:**
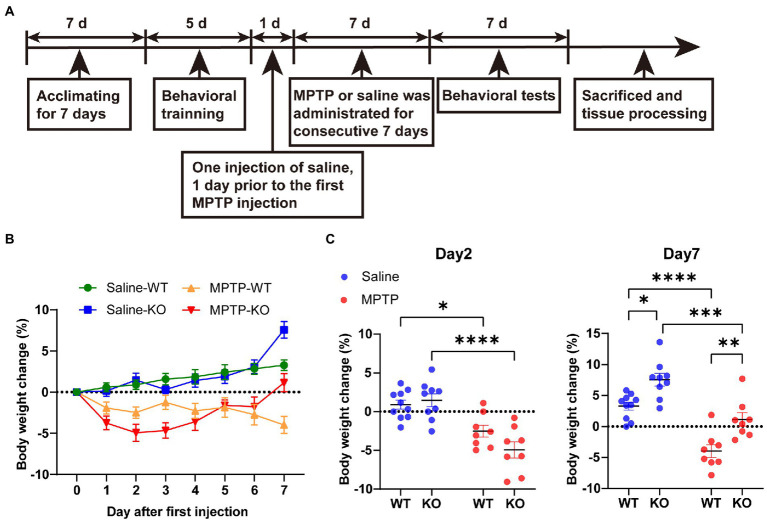
MPTP treatment. **(A)** A schematic diagram of MPTP treatment in *Rab39b* KO and WT mice. **(B)** Percentage of body weight change in MPTP-treated *Rab39b* KO and WT mice or respective saline-treated control mice at different time points after first MPTP injection. **(C)** Percentage of body weight change in MPTP-treated *Rab39b* KO and WT mice or respective saline-treated control mice on days 2 and 7 after first MPTP injection, respectively. Data represent mean ± SEM, *n* = 10 for Saline-WT, *n* = 9 for Saline-KO, and *n* = 8 for both MPTP-WT and MPTP-KO. *p < 0.05, **p < 0.01, ***p < 0.001, ****p < 0.0001, two-way ANOVA followed by Tukey’s *post hoc* test.

*Rab39b* KO mice and their littermate wild-type (WT) control mice were acclimated for 7 days and were subjected to a rotarod training at 10 rpm for 10 min once a day for 5 days for optimum performance (Figure 1A). Mice were then randomly divided into WT control group (saline-WT), *Rab39b* KO control group (saline-KO), MPTP treated WT group (MPTP-WT) and MPTP treated *Rab39b* KO group (MPTP-KO). Mice in each group were intraperitoneally injected with saline once 1 day before the first injection of MPTP. MPTP-WT and MPTP-KO groups were intraperitoneally injected with MPTP (30 mg/kg in 150 μl saline, once a day; MedChemExpress) for 7 consecutive days. Saline-WT and saline-KO groups were given 150 μl saline instead (Figure 1A).

### 2.3. Motor behavioral tests

Mice were tested for motor behaviors after the last injection of MPTP (Figure 1A), and a 30-min adaptation to the experimental environment was provided every day before the test. The experimental environment was soundproof with suitable light intensity and temperature.

#### 2.3.1. Pole test

Mice were placed head-down on top of a vertical wooden pole (55 cm in height and 1 cm in diameter) and the time required to reach the bottom of the pole was measured. The test was repeated three times for each mouse, and the average descending time was calculated for comparison. Two-way ANOVA followed by Tukey’s *post hoc* test was used for the test.

#### 2.3.2. Hanging wire test

A wire with a diameter of 1.5 mm was placed horizontally 40 cm above a thick layer of cotton bedding. Mice were suspended by placing their fore paws on the wire for 30 s and were graded according to the status of their hind paws grasping the wire: both hind paws grasping the wire was scored 3, only one hand paw grasping the wire was scored 2, non-hind paws grasping the wire was scored 1, and mouse falling off the wire was scored 0. Two-way ANOVA followed by Tukey’s *post hoc* test was used for the test.

#### 2.3.3. Rotarod test

The rotarod test was performed as described previously (Niu et al., 2020). Before MPTP injection, mice were trained on a rotating rod at a speed of 10 rpm for 10 min once a day for 5 consecutive days. After the last injection of MPTP, mice were tested on a rotating rod at a constant speed of 10 rpm. Tests were repeated three times at an interval of at least 30 min. The latency to fall off the rotating rod was recorded, and the average of the latencies were calculated and compared. Two-way ANOVA followed by Tukey’s *post hoc* test was used for the test.

#### 2.3.4. Gait analysis

Gait dynamics of mice were captured and analyzed using the DigiGait gait analysis system (Mouse Specifics), as previously described (Quarta et al., 2017; Liu et al., 2019; Wozniak et al., 2019). Mice were placed inside an acrylic compartment (5 cm (W) × 25 cm (L)) with a transparent treadmill belt at the bottom. Each mouse was tested individually on the treadmill; and each paw’s movement was measured. Prior to video recording, mice were allowed to acclimatize for 5 min in the compartment and then run at 10 cm/s for another 5 min to adapt. When mice were stably walking, the belt speed was adjusted and maintained at 25 cm/s. Videos of at least 5 s of uninterrupted mouse running were recorded to provide an adequate number of sequential strides. Once the 5 s videos were obtained, 2.5 s of video length was post-processed and analyzed by the software provided with the DigiGait system. Each video was individually adjusted with a binary threshold adjustment tool to remove any noise and to establish well defined paw areas. Data were manually adjusted to remove any artifacts in the paw area-plots the system. The various measurements were defined or calculated as described below: (1) Stride duration = the stance duration when the paw of a limb was in contact with the treadmill belt; (2) Stride length = speed/stride frequency; and (3) Stride frequency = the number of gait signals over time. Two-way ANOVA followed by Tukey’s *post hoc* test was used for the test.

### 2.4. Immunofluorescence staining

Mice were anesthetized with 1% sodium pentobarbital in saline and perfused with 0.01 M PBS. Whole brain was fixed in 4% paraformaldehyde at 4°C for 24 h, and then dehydrated in 30% sucrose. Tissues were embedded in OCT and 35 μm brain sections were collected by a cryostat microtome (Leica). Mouse brain sections were permeabilized and blocked in buffer composed of 0.2% Triton X-100 and 5% goat serum in PBS for 1 h at room temperature, and then incubated with anti-tyrosine hydroxylase primary antibody (Millipore, ab152, 1: 800), anti-α-synuclein primary antibody (ABclonal, A7215, 1:100), anti-GFAP primary antibody (Proteintech, 16,825-1-AP, 1:200) and anti-IBA1 primary antibody (Wako, 019–19,741, 1:200) overnight at 4°C. After incubating with appropriate fluorescence-conjugated secondary antibodies (Thermo Fisher Scientific, A11008, 1:400) for 1 h at room temperature, z-stack images were obtained using an A1R (Nikon) confocal microscope or an FV1000MPE-B confocal laser scanning biological microscope (OLYMPUS). The number of TH-positive cells in the fluorescence image was counted by particles analysis using Image J. The numbers of α-synuclein-positive cells were counted manually. The mean positive cell number of multiple brain slice images from each mouse was quantified and used for comparison.

### 2.5. Immunoblot analysis

Mouse brains were sliced in pre-cooled brain molds (RWD) and then the substantia nigra was isolated on ice under an optical microscope. Substantia nigra tissues were homogenized and lysed in RIPA lysis buffer (150 mM NaCl, 25 mM Tris–HCl, pH 7.5, 0.1% sodium dodecyl sulfate, and 1% Nonidet P-40) supplemented with the Complete Protease and Phosphatase Inhibitor Cocktail (MedChemExpress). Protein concentration was determined using the Pierce BCA Protein Assay (Thermo Fisher Scientific). Equal amounts of protein lysates were separated by SDS-polyacrylamide gel electrophoresis and transferred to polyvinylidene fluoride membrane. Membrane was blocked with 5% skim milk at room temperature for 1 h, and incubated first with appropriate primary antibody diluent and then with horseradish peroxidase (HRP)-conjugated secondary antibodies. The membranes were incubated with an appropriate amount of ECL (Bio-Rad) solution, which was added dropwise and then imaged with a chemiluminescence imaging system (Azure 300). Protein band intensity was quantified using ImageJ.

### 2.6. Statistical analysis

Statistical analysis was performed using the Prism 8 software (GraphPad). Data represent mean ± standard error of means (SEM). *p* < 0.05 was considered statistically significant.

## 3. Results

### 3.1. MPTP treatment and mouse body weight analysis

Our previous study found that 2-month-old *Rab39b* KO mice showed impaired motor skill learning and decreased midbrain dopamine levels (Niu et al., 2020). To further determine the role of *Rab39b* deficiency in the pathophysiology of PD, we treated *Rab39b* KO and WT control mice with MPTP for 7 consecutive days (Figure 1A). The body weights of mice were recorded right before each injection (Figure 1B). The body weight change in saline-treated WT mice gradually increased during the 7 days. The trend of the body weight change in saline-treated *Rab39b* KO mice was similar to that of saline-treated WT mice (Figure 1B). But on day 7, the body weight gain was much more in saline-treated *Rab39b* KO mice than in saline-treated WT mice (Figure 1C). On the other hand, the body weight change in WT and *Rab39b* KO mice treated with MPTP decreased significantly after the first injection, when compared to respective saline-treated control group. The body weight loss in WT mice kept increasing throughout the MPTP treatment. However, after the first three injections of MPTP, *Rab39b* KO mice loss more weight than WT mice with no significant difference, then the weight of *Rab39b* KO mice began to recover, and after the last injection the weight of *Rab39b* KO mice even exceeded that before treatment. In addition, the weight gain in *Rab39b* KO mice was also higher than that of WT group after the seventh injection of saline (Figures 1B,C).

### 3.2. Loss of *Rab39b* does not alter MPTP-induced motor behavior impairment

**Figure 2:**
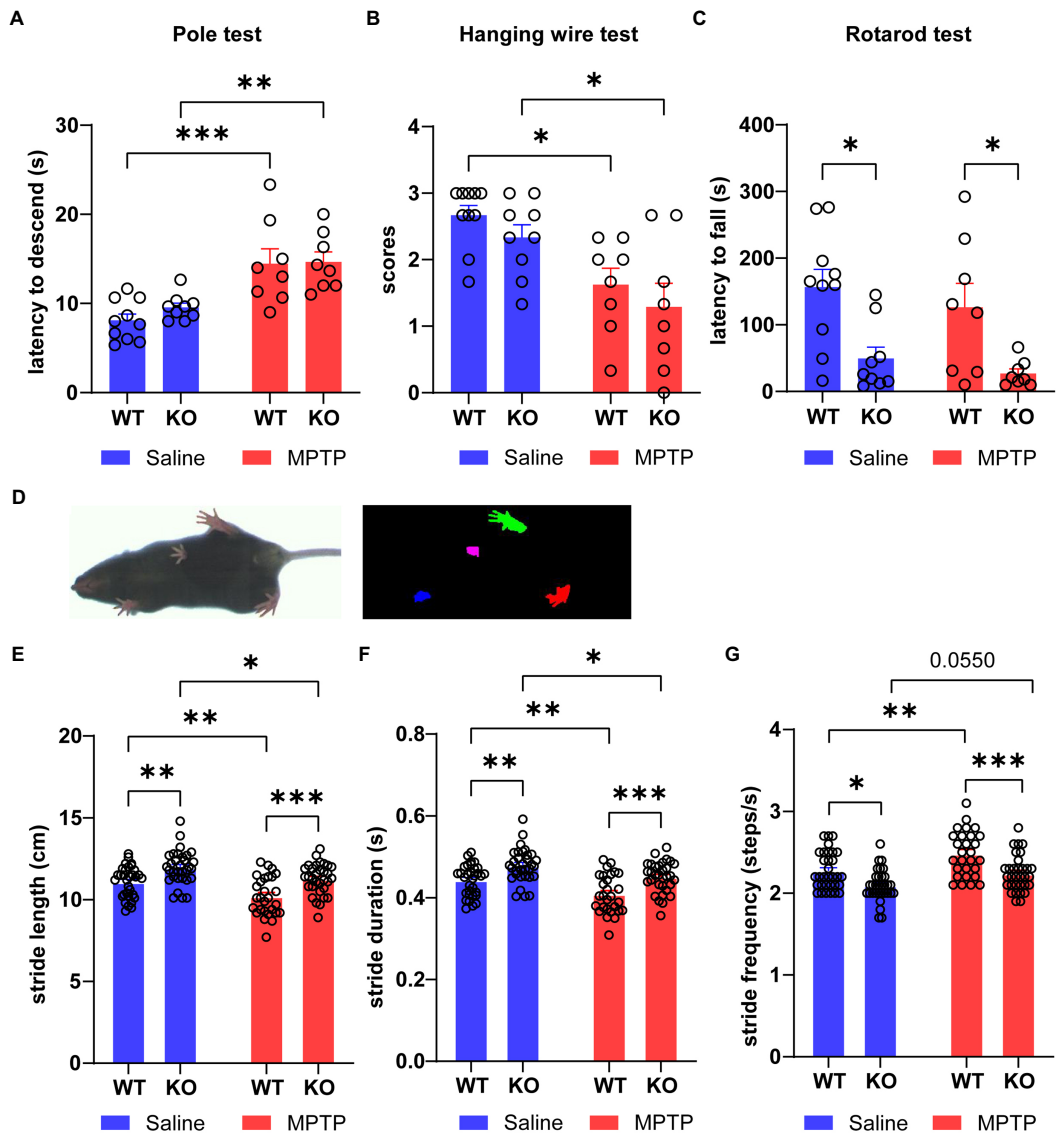
Loss of *Rab39b* does not alter motor behavior impairment in MPTP-induced mice. **(A)** Latency of descending from the top to the bottom in MPTP-treated *Rab39b* KO and WT mice or respective saline-treated control mice was recorded in pole test for comparison. *n* = 10 for Saline-WT, *n* = 9 for Saline-KO, and *n* = 8 for both MPTP-WT and MPTP-KO. **(B)** Hanging wire test was performed in MPTP-treated *Rab39b* KO and WT mice or respective saline-treated control mice and the scores were calculated for comparison. *n* = 10 for Saline-WT, *n* = 9 for Saline-KO, and *n* = 8 for both MPTP-WT and MPTP-KO. **(C)** MPTP-treated *Rab39b* KO and WT mice or respective saline-treated control mice were subjected to the rotarod test and the average latency to fall was calculated for comparison. *n* = 10 for Saline-WT, *n* = 9 for Saline-KO, and *n* = 8 for both MPTP-WT and MPTP-KO. **(D)** Representative image of a mouse subjected to video recording of gait analysis (left) and digital image of the ventral surface of the gait footprints during the analysis (right). **(E-G)** The stride length, stride duration, and stride frequency in MPTP-treated *Rab39b* KO and WT mice or respective saline-treated control mice. *n* = 32 paws from 8 mice for Saline-WT, Saline-KO and MPTP-KO, and *n* = 28 paws from 7 mice for MPTP-WT. Data represent mean ± SEM, *p < 0.05, **p < 0.01, ***p < 0.001, two-way ANOVA followed by Tukey’s *post hoc* test.

After the induction of MPTP, the mice were tested for motor behaviors. In the pole test, the latency to descend from the top to the bottom in MPTP-treated WT and *Rab39b* KO mice was significantly longer than that in respective saline-treated control group. However, there was no significant difference between *Rab39b* KO and WT mice in either MPTP or saline treatment group (Figure 2A). Similarly, in the hanging wire test, the scores in MPTP-treated WT and *Rab39b* KO mice were significantly lower than those in corresponding saline-treated mice, but there was no significant difference between *Rab39b* KO and WT mice in either MPTP or saline treatment group (Figure 2B). In the rotarod test, although MPTP induction did not significantly change the motor ability of *Rab39b* KO mice, the average latency to fall in *Rab39b* KO mice was significantly reduced when compared to corresponding WT mice in both MPTP and saline treatment groups (Figure 2C). In addition, mice were subjected to gait analysis tests (Figure 2D). Consistently, MPTP-treated WT and *Rab39b* KO mice walked with significantly shorter stride time and stride length, as well as higher stride frequency, compared to the respective saline-treated control group. Furthermore, compared to the corresponding WT mice in MPTP and saline treatment groups, both *Rab39b* KO mice showed longer stride time and stride length, as well as lower stride frequency (Figures 2E–G). These results suggest that MPTP treatment causes motor activity impairment in mice, but loss of *Rab39b* does not alter MPTP-induced motor behavior impairment.

### 3.3. *Rab39b* deficiency does not alter MPTP-induced pathology of PD in SNpc

**Figure 3:**
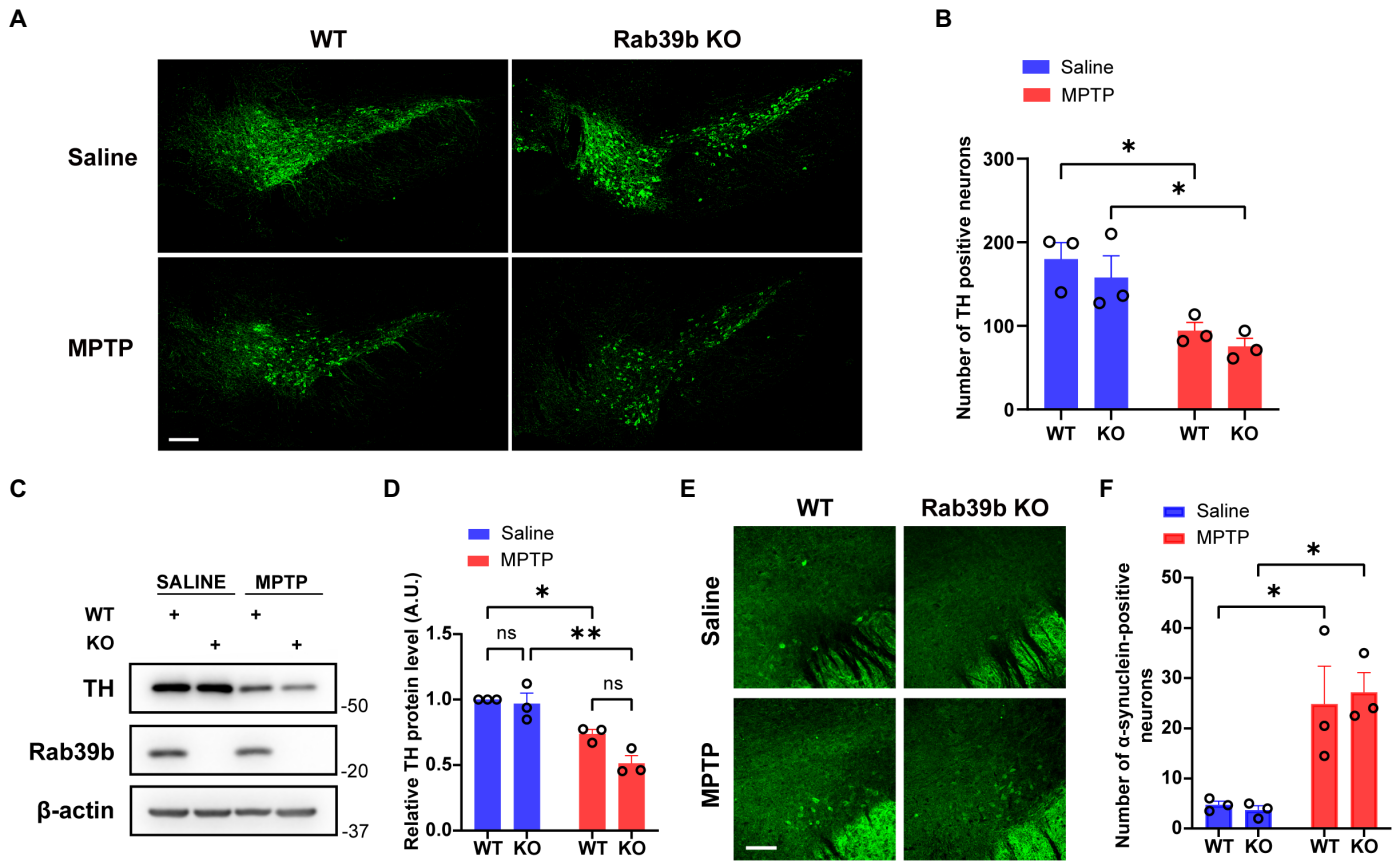
Loss of *Rab39b* does not alter MPTP-induced pathology of PD in SNpc. **(A)** Representative images of TH immunostaining of SNpc in MPTP-treated *Rab39b* KO and WT mice or respective saline-treated control mice. Scale bar, 200 μm. **(B)** Quantitative analysis of TH positive cell numbers in **(A)**. *n* = 3 mice per group, average of 2–4 brain slices from each mouse. **(C)** Western blotting of proteins in substantia nigra tissue lysates of mice. **(D)** Protein levels were quantified and normalized to those of β-actin for comparison. *n* = 3 mice per group. **(E)** Representative images of α-synuclein immunostaining of substantia nigra in MPTP-treated *Rab39b* KO and WT mice or respective saline-treated control mice. Scale bar, 80 μm. **(F)** Quantitative analysis of α-synuclein positive cell numbers in **(E)**. *n* = 3 mice per group, average of 2–4 brain slices from each mouse. Data represent mean ± SEM, ns: not significant, *p < 0.05, **p < 0.01, two-way ANOVA followed by Tukey’s *post hoc* test.

Dopaminergic neurons in SNpc are subjected to progressive loss in PD. Therefore, we performed immunofluorescence staining to quantitatively analyze the number of tyrosine hydroxylase (TH)-positive dopaminergic neurons in SNpc of mice. The number of TH-positive cells in SNpc of MPTP-treated WT and *Rab39b* KO mice was significantly lower than that of respective saline-treated mice, the number of TH-positive cells in SNpc was not significantly changed between WT and *Rab39b* KO mice in either MPTP or saline treatment group (Figures 3A,B). Meanwhile, western blotting of the substantia nigra showed significant decrease of TH protein levels in MPTP-treated WT and *Rab39b* KO mice compared to respective saline-treated mice, and the TH protein level was not significantly changed between WT and *Rab39b* KO mice in either MPTP or saline treatment group (Figures 3C,D). In addition, we quantified α-synuclein-positive neurons in the substantia nigra region by immunofluorescence. In the saline control group, most of the neuronal bodies lacked detectable α-synuclein staining, whereas fluorescence was mainly manifested in the neuronal fibers. Compared to the respective saline control group, the number of α-synuclein-positive cells in substantia nigra of MPTP-treated WT and *Rab39b* KO mice was significantly elevated (Figures 3E,F). However, *Rab39b* KO mice showed no change compared to WT levels under the MPTP-induced pathological context. These results suggest that MPTP induction decreases the number of dopaminergic neurons and increases α-synuclein reactivity of neurons in substantia nigra of mice, but deletion of RAB39B does not further affect these pathologies of PD induced by MPTP.

### 3.4. Loss of *Rab39b* does not alter MPTP-induced gliosis

**Figure 4:**
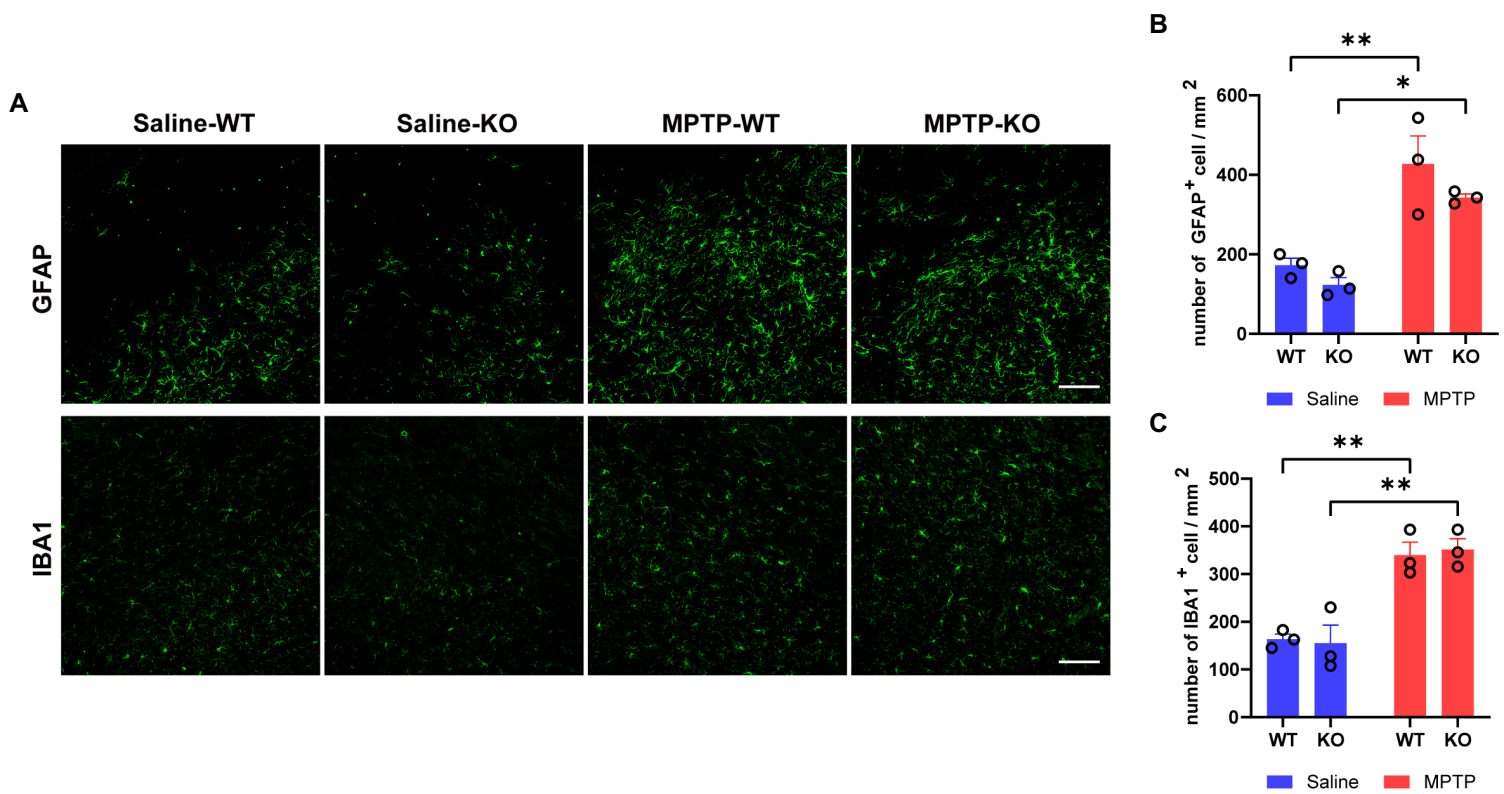
Loss of *Rab39b* does not alter reactive gliosis in SNpc of MPTP-induced mice. **(A)** Representative images of GFAP and IBA1 immunostaining of SNpc in MPTP-treated *Rab39b* KO and WT mice or respective saline-treated control mice. Scale bars, 100 μm. **(B)** Quantitative analysis of GFAP-positive cells in **(A)**. **(C)** Quantitative analysis of IBA1-positive cells in **(A)**. *n* = 3 mice per group, average of 2–3 brain slices from each mouse. Data represent mean ± SEM, *p < 0.05, **p < 0.01, two-way ANOVA followed by Tukey’s *post hoc* test.

We then studied the role of *Rab39b* deficiency in MPTP-induced gliosis in mice. Immunofluorescence staining and quantitative analysis demonstrated that the number of GFAP-positive astrocytes and IBA1-positive microglia was significantly increased in SNpc of MPTP-treated WT and *Rab39b* KO mice when compared to respective saline-treated mice, but there was no significant difference between WT and *Rab39b* KO mice in either MPTP or saline treatment group (Figures 4A–C). These results suggest that MPTP toxicity causes gliosis of astrocytes and microglia, but loss of *Rab39b* does not affect gliosis, nor does it alter glial activation in response to MPTP toxicity.

## 4. Discussion

Recent studies have reported an increasing number of *RAB39B* mutations associated with early-onset PD as well as Lewy body diseases (LBDs) (Wilson et al., 2014; Lesage et al., 2015; Mata et al., 2015; Güldner et al., 2016; Shi et al., 2016; Ciammola et al., 2017). Most of these mutations cause the loss of RAB39B protein function. But the molecular mechanism underlying PD pathogenesis caused by *RAB39B* mutations remains to be clarified. Previous studies have shown that loss of RAB39B compromises motor learning but does not affect mobility in 2-month-old mice (Niu et al., 2020; Zhang et al., 2020). Herein, we constructed a PD model induced by intraperitoneal injection of MPTP in *Rab39b* KO mice to explore the possible consequences of RAB39B deficiency in PD pathology. *RAB39B* is an X-linked gene and EOPD associated with RAB39B mutations are more common in men than women, so we focused on male mice in this study.

MPTP treatment is a classic and widely used method to generate animal model of PD (Jackson-Lewis and Przedborski, 2007). MPTP can pass through the blood–brain barrier (BBB) and is metabolized by astrocytic monoamine oxidase-B (MAO-B) to form the active toxicant 1-methyl-4-phenylpyridinium (MPP^+^), which accumulates in the mitochondrial matrix and inhibits complex I of the respiratory chain, resulting in degeneration and death of nigra-striatal dopaminergic neurons (Heikkila et al., 1984; Di Monte et al., 1991, 1992). During the MPTP treatment, we found a significant and persistent decrease in the body weight in MPTP-treated mice compared to respective control mice after the first injection. Interestingly, although the weight loss of *Rab39b* KO mice was more significant than that of WT mice after the first three injections of MPTP, the weight of *Rab39b* KO mice began to recover and returned to the level before MPTP treatment after the last injection. In previous studies, *Rab39b* KO mice showed some weight loss compared to littermate control WT at 2 months of age or throughout the early stage of growth (P7-P90), regardless of the mouse strains (Niu et al., 2020; Mignogna et al., 2021). The loss of body weight in *Rab39b* KO mice could be attributed to a significant reduction in the volume of abdominal adipose tissue, whereas there were no significant differences in the weight of organs, food and water intake, body temperature, and glucose consumption in each brain region between genotypes (Mignogna et al., 2021). Lack of activity caused by MPTP treatment translate into reduced food and water intake and a possible slight weight loss (Jackson-Lewis and Przedborski, 2007). At the beginning of intraperitoneal injection of MPTP (the first three injections), *Rab39b* KO mice with less abdominal adipose tissue may be more sensitive to drug reactivity. Given that saline-injected *Rab39b* KO mice on the seventh day also gained more weight than saline-injected WT mice, this abnormal weight gain may be due to that *Rab39b* KO mice are still in a growth and development stage with unknown reason. Future investigation on the water and food intake and metabolic indexes of mice during injection period may help solve this puzzle.

The results of pole test and hanging wire test showed that MPTP-treated mice had deficits in motor abilities, but loss of RAB39B did not exacerbate these impairments. In the rotarod test, the latency to fall from the rotating rod in *Rab39b* KO mice was significantly lower than that in corresponding WT mice in both MPTP and saline treatment groups, but there was no difference between MPTP-and saline-treated *Rab39b* KO mice. These results are consistent with previous studies suggesting that loss of RAB39B impairs motor ability learning (Niu et al., 2020; Zhang et al., 2020), and further indicate that loss of RAB39B does not alter motor deficits upon MPTP toxicity. In gait analysis test, we found that both saline-and MPTP-treated Rab39B KO mice exhibited increased stride length and duration and decreased stride frequency when compared to respective WT controls. While upon MPTP treatment, both WT and *Rab39b* KO mice had decreased stride length and duration and increased stride frequency; and this is consistent with the phenotype of MPTP-induced mice reported by Liu et al. (2019). These findings further support that RAB39B deficiency does not affect MPTP-induced motor deficits in mice. Moreover, our results revealed that RAB39B deficiency had no effect on dopaminergic neuronal loss and neuroinflammatory response in SNpc of MPTP-induced mice. Together, in the current study, we demonstrate that RAB39B deficiency does not contribute to PD through compromising dopaminergic neurons in MPTP-induced PD mouse model.

The α-synuclein protein is highly expressed in brain and is a primary component of Lewy bodies in PD (Goedert et al., 2017; Khan and Khan, 2022). Missense mutations, as well as multiplications of the gene encoding α-synuclein (*SNCA*) are directly related to familial PD (Menšíková et al., 2022). RAB39B was found to be involved in the regulation of α-synuclein homeostasis in some previous studies (Wilson et al., 2014; Gonçalves et al., 2016). Wilson et al. reported that the loss of RAB39B in primary hippocampal neurons and P19 neuroblastoma reduced steady-state levels of α-synuclein (Wilson et al., 2014). Subsequently, Gonçalves et al. found that the RAB39B silencing promoted the oligomerization and aggregation of α-synuclein independent of the levels of α-synuclein in human H4 neuroglial cells (Gonçalves et al., 2016). Herein, we found that MPTP treatment significantly increased the number of α-synuclein-positive cells in substantia nigra of both WT and *Rab39b* KO mice; and these results are consistent with previous studies showing an up-regulation of α-synuclein in dopaminergic neurons in substantia nigra after injection of MPTP in both mice and non-human primates (Vila et al., 2000; Purisai et al., 2005). However, loss of RAB39B did not further affect the number of a-synuclein-positive cells exposed to MPTP toxicity. Future studies to explore whether RAB39B deficiency contributes to α-synuclein pathology *in vivo*, using Lewy body pathological models such as the A53T transgenic mice (Koprich et al., 2017) may help determine the mechanism underlying the pathogenesis of PD caused by *RAB39B* mutations.

## Data availability statement

The original contributions presented in the study are included in the article/supplementary material, further inquiries can be directed to the corresponding author.

## Ethics statement

The animal study was reviewed and approved by Animal Ethics Committee of Xiamen University.

## Author contributions

ZW, Y-wZ, and XZ designed the research and wrote the manuscript. ZW and DY performed most molecular and animal experiments. YJ, YW, and MN helped with animal experiments. CW, HL, HX, and JL made intellectual contributions. All authors reviewed the manuscript.

## Funding

This work was supported by grants from National Natural Science Foundation of China (81771377, U21A20361 and 82130039 to Y-wZ).

## Conflict of interest

The authors declare that the research was conducted in the absence of any commercial or financial relationships that could be construed as a potential conflict of interest.

## Publisher’s note

All claims expressed in this article are solely those of the authors and do not necessarily represent those of their affiliated organizations, or those of the publisher, the editors and the reviewers. Any product that may be evaluated in this article, or claim that may be made by its manufacturer, is not guaranteed or endorsed by the publisher.
